# Effect of Modifying the Membrane Surface with Microcapsules on the Flow Field for a Cross-Flow Membrane Setup: A CFD Study

**DOI:** 10.3390/membranes11080555

**Published:** 2021-07-22

**Authors:** Sebastian Osterroth, Christian Neumann, Michael Weiß, Uwe Maurieschat, Alexandra Latnikova, Stefan Rief

**Affiliations:** 1 Fraunhofer Institute for Industrial Mathematics ITWM, 67663 Kaiserslautern, Germany; stefan.rief@itwm.fraunhofer.de; 2 Fraunhofer Institute for Applied Polymer Research IAP, 14476 Potsdam, Germany; christian.neumann@iap.fraunhofer.de (C.N.); michael.weiss@iap.fraunhofer.de (M.W.); alexandra.latnikova@iap.fraunhofer.de (A.L.); 3 Fraunhofer Institute for Manufacturing Technology and Advanced Materials IFAM, 28359 Bremen, Germany; uwe.maurieschat@ifam.fraunhofer.de

**Keywords:** membrane biofouling, simulation, virtual material design, structuring, microcapsules, adhesive microdrops

## Abstract

In this study, the attachment of microcapsules on the membrane surface and its influence on the flow field for a cross-flow membrane setup are investigated. The microcapsules were placed on the top layer of the membrane. The overall purpose of this modification was the prevention of membrane biofouling. Therefore, in a first step, the influence of such a combination on the fluid flow was investigated using computational fluid dynamics (CFD). Here, different properties, which are discussed as indicators for biofouling in the literature, were considered. In parallel, different fixation strategies for the microcapsules were experimentally tested. Two different methods to add the microcapsules were identified and further investigated. In the first method, the microcapsules are glued to the membrane surface, whereas in the second method, the microcapsules are added during the membrane fabrication. The different membrane modifications were studied and compared using CFD. Therefore, virtual geometries mimicking the real ones were created. An idealized virtual geometry was added to the comparison. Results from the simulation were fed back to the experiments to optimize the combined membrane. For the presented setup, it is shown that the glued configuration provides a lower transmembrane pressure than the configuration where microcapsules are added during fabrication.

## 1. Introduction

Over the past few decades, a great variety of membranes for the filtration of solids, purification of gases, purification of fluids, or control of process parameters have been developed. Depending on the separation process and the related conditions, different materials and morphologies of membranes can be used [1]. The great variety of membranes led to different membrane classifications: dense or porous; organic or inorganic; biobased or synthetic; flat sheet or hollow fiber; symmetric or asymmetric; homogeneous or heterogeneous. However, there is one major drawback that almost all membranes have in common: they show a decrease of permeability over time due to membrane fouling (e.g., [2,3,4]). Especially biofouling is a major problem in water treatment (e.g., [4,5,6]). In the case of biofouling, a low-permeable biofilm grows on the surface of the membrane. Depending on the operation mode, this leads to a decline of the flux through the membrane or an increase of the differential pressure. The growth and the structure of such a biofilm depend on various factors such as the type of microorganism, the surface material and surface structure of the membrane, or the quality of the feed water [4]. One conventional strategy to prevent biofouling is to dose biocides or antimicrobial substances continuously with the feed water. The efficiency of this method depends on factors such as contact time, concentration, or pH. However, the continuous addition of biocide might lead to unsustainable resource usage, which causes an increase of the cost of the process, as well as environmental, ecological, or toxicological problems. Therefore, one important utility to reduce these disadvantages is the continuous monitoring of the separation process including the biofouling process itself (e.g., [7]). Then, based on the monitoring, appropriate countermeasures can be taken. Shock or pulse dosing is used as a strategy to reduce the amount of biocide (e.g., [8,9]).

Another method to prevent biofouling is the modification of the membrane surface by choosing a specific material, a chemical modification of the surface (e.g., [6,10,11]), or by the fabrication of nanocomposites (see, e.g., [3] for an overview).

In the past, several ideas to use microparticles or microcapsules to avoid fouling were investigated. In 1985, a patent was published [12], in which microcapsules with antifouling compounds were added to the feed. The microcapsules are transported towards the membrane (together with the fouling material) and deposited there. The slowly released compounds from the microcapsules can directly act at the appropriate site, where they should reduce membrane fouling by reducing the thickness of the fouling layer. In [13], the authors described microparticles with a functionalized surface that carries antimicrobial molecules to reduce biofouling. The loaded microparticles were added to the solution (stationary), and the survival rate of the microorganisms as a measure of the biofouling was decreased. In these works, the biofouling behavior in channels or pipes was investigated. In other cases, the microcapsules are included or added at the surface of a device as is for example the case for coatings (e.g., [14]).

Successful integration of the microcapsules into the membranes for other purposes was already demonstrated in the literature. In [15,16], microcapsules were embedded within the membrane to provide a self-healing feature when the active layer was damaged during installation or operation. For enhancing CO_2_ separation, composite microcapsules were integrated into mixed matrix membranes in [17]. In such a setup, the microcapsules are used as fillers. Due to the surface structure of the composite microcapsules, the transmembrane mass transfer resistance is reduced. In [18], polymeric microcapsules were incorporated into the membrane in order to improve the water retention properties of composite membranes, which also led to increased conductivity. Microcapsules were added as mass retention reservoirs to polymer membranes in [19]. Recently, many studies have been performed on the integration of nanocapsules into membranes [20,21,22,23]. For example, in [22], silver nanoparticles were sandwiched between graphene oxide layers, and this nanosheet was added to the membrane surface by a polymerization process. Their results showed that biofouling was reduced. In [23], different methods to modify the surface of ceramic membranes were presented.

Microencapsulation is a widely applied technology, which implies the formulation of various functional substances in the form of composite microcapsules with a defined structure. Embedding of biocides into polymeric shells allows the realization of controlled release profiles (e.g., [14,24,25]).

The idea of this study is to locate the microcapsules containing biocide onto the membrane surface instead of integrating them into the bulk of the membrane. Thus, the microcapsules can release the biocide at the places where it is needed. To the best of the authors’ knowledge, such a study has not been performed before.

The usage of microcapsules and their fixation onto the membrane surface have multiple advantages:

1. Microcapsules are placed at the region (membrane surface) where membrane biofouling mainly takes place;
2. The concentration of biocide can be significantly reduced due to the controlled release from the microcapsules. Adding microcapsules in the feed requires a much higher concentration since only part of the inflow reaches the membrane (at least in a cross-flow setup). In comparison, a lower number of microcapsules and thus a lower total biocide concentration are needed when the microcapsules are directly fixed at the membrane surface;
3. The number of microcapsules at the membrane surface can be adjusted to higher or lower values in accordance with the application scenario;
4. The microcapsules might be equipped with a stimulus-responsive mechanism to release their contents, for example when a specific concentration of a target substance is reached in the biofouling layer;
5. In the case of pore blocking caused by biofouling, the local concentration of the biocide will increase automatically due to the proximity of microcapsules to the membrane surface.

Despite all the advantages, the production of such a sophisticated combined microcapsule-membrane without compromising the membrane performance is a challenge. Here, mainly two difficulties arise. First, the characteristics of the membrane (and especially the active layer) should be influenced as little as possible by the addition of the microcapsules. Second, it has to be ensured that the microcapsules are located at the surface of the membrane. Therefore, conventional strategies for microcapsule fixation such as the ones used for textile finishing are not appropriate because the nanopores of the membrane might be locked [26]. Chemical cross-linking between microcapsules and the membrane is also unfavored due to the high costs of functionalization [27]. While keeping these considerations in mind, two different methods for the fixation of microcapsules onto the membrane surface were developed in this study. In one method, the microcapsules are combined with membranes during the membrane production, resulting in a microcapsule membrane with a half-sphere morphology [28]. In the other method, the fixation of microcapsules onto ready membranes is realized using a microdispensing technology, which allows for the application of single glue dots without clogging all membrane pores followed by microcapsule fixation.

To assist the choice of a suitable fixation technique and to further answer design questions, mathematical modeling and simulation were used. Simulation is a useful tool to design and optimize membrane processes. The performance of a membrane separation process is highly influenced by the membrane surface area and the surrounding fluid dynamics. Thus, it is necessary to identify the most influencing parameters. Both simulations and experiments are an iterative way to obtain an enhanced setup. However, simulations enable testing many and maybe unusual scenarios, for example by setting parameters to values that cannot yet be realized in an experimental setup. Since the setup at hand is new, simulations are used to obtain first insights into the influence of the microcapsules and their configurations on the flow field, which is an indicator of the biofouling behavior. This information is used to suggest the design of a promising setup for further experimental investigations.

The application of computational fluid dynamics (CFD) for a broad variety of problems in membrane processes has been often described in the literature. Ghidossi and co-authors conducted a literature review [29] and emphasized four main reasons and tasks for using computational fluid dynamics (CFD): (i) testing new membrane materials, (ii) using different pore sizes, (iii) determining conditions for optimal selectivity, and (iv) finding conditions to minimize biofouling. This study focuses on the third and the fourth point. In another review, Fimbres-Weihs and Wiley [30] focused on the influence of spacers and compared 2D and 3D models and their numerical solutions. The simulation of pressure-driven membrane filtration was reviewed by Keir and Jegatheesan [31]. A review of modeling approaches for spiral wound membrane desalination modules was conducted by Karabelas et al. [32]. The authors summarized several modeling approaches and spacer geometries. An overview of CFD simulations for membrane distillation processes was conducted by Shirazi et al. [33]. A recent review of mathematical models for flow in membrane-based filtration systems was given by Parasyris et al. [34]. They distinguished three categories of models based on the treatment of the porous membrane: (i) microscopic, (ii) reduced, and (iii) mesoscopic models. In the spirit of this classification, the model used in the present work is mesoscopic since the membrane is treated as a uniform porous medium and Darcy’s law is considered. In [35], the scaling of membrane processes and the use in simulation tools were discussed.

From a mathematical point of view, the utilized numerical methods can be divided into finite volume (FVM) and finite element (FEM) methods. General differences between the two methods can be found in [36]. FVMs were used in [37,38] utilizing OpenFOAM and in [39,40,41,42] utilizing ANSYS Fluent. FEMs were considered in [34,43] utilizing COMSOL. As already mentioned, CFD is mostly used to analyze the geometry. In [37], the layering of fibers as spacers was investigated. In [43], a two-dimensional model for vacuum membrane distillation was used to perform a design study. In [38], the effect of the module geometry on the flow field was investigated. For a hollow fiber setup, the alignment of the flow and the position of the outlet were adjusted. Moreover, the authors of [40,44] investigated the design and the influence of spacers on the flow field. The design of a side-flow filtration module in a bioreactor was investigated in [45]. The influence of several obstacles on the flow field and mass transfer was studied in [46]. Oscillatory flow and enhanced slip velocity were investigated in [42].

One specific characteristic, which is widely studied using CFD, is the wall shear stress. It is reported to be an indicator for the reduction of membrane biofouling (e.g., [47]). A review on this problem for pressure-driven membrane systems was given by [48]. Many studies tried to enhance the wall shear stress to reduce the accumulation at the surface of the membrane [39,47,49]. In [50], the wall shear stress for hollow fiber membrane modules was investigated and different module configurations were compared. It was shown that a tangential inlet/outlet configuration leads to higher wall shear stresses.

This likely incomplete list shows that CFD methods are widely used for design studies investigating several aspects of membrane processes.

The article at hand focuses on methods of membrane functionalization with microcapsules. The production of membranes with various types of modification, as well as the experimental testing of the resulting membranes’ performance are complex and time and resource consuming. Therefore, a dual approach is followed. The experimental work on the microcapsule fixation and simulations are used side by side to directly incorporate findings from the simulations in the membrane design and vice versa. The simulations started with an idealized setup (compare Section 4.2) and then were closely related to the realizable configurations from the experiments (Section 4.3 and Section 4.4). For these configurations, the influence of the microcapsules on the flow was studied using CFD. Since the microcapsules are obstacles to the flow, they provide an additional resistance. Additionally, they reduce the open surface of the membrane, yielding an increase of the transmembrane pressure. Therefore, the difference between the setup with and without microcapsules was determined. Furthermore, it was investigated how different geometrical arrangements of the microcapsules influence the result. To gain insights into potential membrane biofouling, the vorticity and the shear stresses were visualized and rated since they are considered as an indicator of membrane biofouling.

The article is organized as follows: First, the mathematical model is introduced. Then, the cross-flow setup is shown, and further details on the membrane components are presented. Afterward, three setups for the fixation and multiple configurations of the microcapsules on the membrane surface are investigated. The article closes with conclusions and an outlook.

## 2. Mathematical Model

In the following, the mathematical description of a flow in a bounded domain in a cross-flow setup is presented. The formulation closely follows the description of the mesoscopic model in [34].

**Figure 1:**
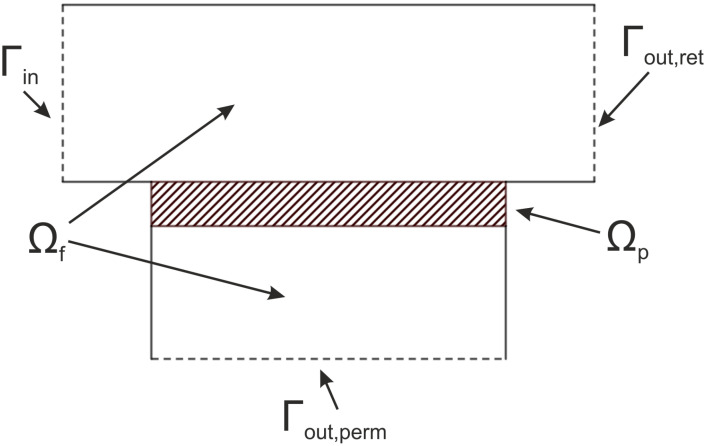
A schematic view of a vertical cross-section of the computational domain. The hatched part depicts the membrane.

Consider a domain $\Omega =\Omega_{f}\cup \Omega_{p}\subset R^{3}$ with spatial coordinate $x=(x_{1},x_{2},x_{3})$. The domain is divided into two parts, the membrane (porous) part $\Omega_{p}$ and the free flow part $\Omega_{f}$ (on both sides of the membrane). The boundary Γ of the domain Ω is decomposed into several parts as shown in the 2D projection in Figure 1. The fluid enters the domain using the feed inlet, which is denoted by $\Gamma_{in}$. The outflow is split into two parts. The fluid either leaves the domain via the retentate outlet denoted by $\Gamma_{out,ret}$ or the permeate outlet denoted by $\Gamma_{out,perm}$, respectively. The boundaries in the projection plane (front or back, respectively) are considered as symmetric $\Gamma_{sym}$. The remaining parts of the boundary are solid walls, denoted by $\Gamma_{wall}$.

The fluid under consideration is water, but the proposed simulation model can be applied in general to laminar, incompressible, and isothermal flow regimes. The constant fluid density is denoted by $\rho_{f}$ and the dynamic viscosity by μ, respectively. Furthermore, the membrane, or in general the porous medium, is considered to be homogeneous and isotropic with effective properties, e.g., permeability *K*. Such a flow can be described using a velocity field *u* and a pressure distribution *p*. The unstationary incompressible Navier–Stokes–Brinkman equations read [51] (see also [34]):

(1a) $$\begin{array}{cccc} \nabla \;\cdot \;u & =0, & \; & x\in \Omega , \end{array}$$

(1b) $$\begin{array}{cccc} \rho_{f}\left(\frac{\partial u}{\partial t}+\left(u\;\cdot \;\nabla \right)u\right)-\mu_{eff}\Delta u+\mu K^{-1}u & =-\nabla p, & \; & x\in \Omega . \end{array}$$

The first equation describes the conservation of mass and the second the conservation of momentum. From a theoretical point of view, an effective viscosity $\mu_{eff}$ has to be added. It is equal to the fluid viscosity μ in the free flow part $\Omega_{f}$, and in the porous part, its value $\mu_{p}$ needs to be estimated by experiments. As was explained in [34], there is up to now no consensus on how to determine $\mu_{p}$. Therefore, $\mu_{p}\approx \mu$ is an accepted assumption. Note that the equations are valid in both the free flow and the membrane part of the domain since in the free flow part, $\Omega_{f}$, the inverse of the permeability is set to zero, i.e., $K^{-1}=0$. In general, such a formulation is known as a one-domain approach. However, in the membrane part of the domain, the equations approximate Darcy’s law since the inverse of the (low) permeability is the dominating term there. Thus, one obtains the relation:

(2) $$u=-\frac{K}{\mu }\nabla p.$$

To be more precise, if the main flow direction is the $x_{3}$-direction, the average flow speed $u_{\mathrm{average}}$ is given as:

(3) $$u_{\mathrm{average}}=\frac{Q}{A}=-\frac{K}{\mu }\frac{\partial p}{\partial x_{3}},$$

where *Q* is the flow rate and *A* the cross-sectional area of the membrane. Performing integration on both sides, the integral form of Darcy’s law,

(4) $$Q=\frac{KA}{\mu L}\left(p_{\mathrm{downstream}}-p_{\mathrm{upstream}}\right),$$

can be deduced, where *L* denotes the thickness of the membrane.

The boundary conditions are chosen in a way such that the fluid flux over the membrane is kept constant. An (constant) inflow velocity $u_{in}$ (or equivalently a given flow rate $Q_{in}$) is described at $\Gamma_{in}$. At the permeate outlet, also the velocity $u_{out}$ (or flow rate $Q_{out}$) is fixed. This leads to a constant flux through the membrane, and it is possible to describe how the flux is split into a retentate and permeate portion. At the retentate outlet, a fixed pressure value is prescribed. Symmetry conditions for the normal component of the velocity and the pressure gradient are used at $\Gamma_{sym}$. At the solid walls $\Gamma_{wall}$, no-slip boundary conditions are prescribed. Thus, the boundary conditions can be summarized as follows:

(5a) $$\begin{array}{cccccc} u(x,t) & =u_{in}, & \; & \frac{\partial p}{\partial n}\left(x,t\right) & =0, & \;x\in \Gamma_{in}, \end{array}$$

(5b) $$\begin{array}{cccccc} \frac{\partial u}{\partial n}\left(x,t\right) & =0, & \; & p(x,t) & =0, & \;x\in \Gamma_{out,ret}, \end{array}$$

(5c) $$\begin{array}{cccccc} u(x,t) & =u_{out}, & \; & \frac{\partial p}{\partial n}\left(x,t\right) & =0, & \;x\in \Gamma_{out,perm}, \end{array}$$

(5d) $$\begin{array}{cccccc} u(x,t)\;\cdot \;n(x,t) & =0, & \; & \frac{\partial p}{\partial n}\left(x,t\right) & =0, & \;x\in \Gamma_{sym}, \end{array}$$

(5e) $$\begin{array}{cccccc} u(x,t) & =0, & \; & \frac{\partial p}{\partial n}\left(x,t\right) & =0, & \;x\in \Gamma_{wall}. \end{array}$$

Here, *n* denotes the outer unit normal vector of the boundary. Below, the normal vectors are also indicated as $n_{in}$ and $n_{out,perm}$ to denote the outer unit normal vector at the inlet and the permeate outlet, respectively. At the initial stage, the fluid is described by the following conditions:

(6) $$u\left(x,0\right)=u_{0},\;p\left(x,0\right)=p_{0},\;x\in \Omega .$$

With the above set of Equations (1), (2) and (6), the fluid flow in the cross-flow setup is described. This formulation is used in the following for the simulation. When microcapsules are inserted into the channel on the surface of the membrane, their surface is considered as an additional part of $\Gamma_{wall}$.

Another quantity to describe the flow is the vorticity ω [52]. It is a pseudovector field and defined as the curl of the velocity field:

(7) ω=∇×u.

It describes the local spinning motion or angular velocity in a flow and might be seen as an indicator of local vortices. Since it is based on the velocity gradient, it can give pieces of useful information on the mixing behavior in the fluid. As stated in [41], “*higher velocity variations near the membrane can be useful in enhancing heat and mass transfer*.”

The wall shear stress τ at a surface orthogonal to the $x_{3}$-axis is defined as:

$$\tau =\mu \frac{\partial u_{\mathrm{along}}}{\partial x_{3}},$$

where $u_{\mathrm{along}}$ is the velocity along the surface (compare, e.g., [39] with a different sign). In a more general form, it can be defined as:

(8) $$\tau =\mu {\left|\nabla u\right|}_{\mathrm{wall},\tan\mathrm{gent}}$$

with ${\left|\nabla u\right|}_{\mathrm{wall},\mathrm{tangent}}$ being the magnitude of the tangential components of the velocity at the wall [53]. As can be seen, the shear stress is proportional to the velocity gradient.

## 3. Cross-Flow Setup

**Figure 2:**
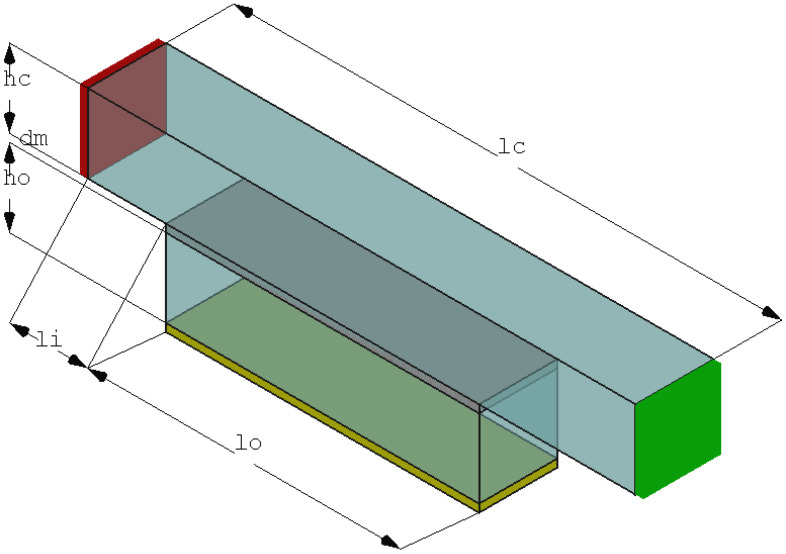
Computational setup: membrane in gray, feed inlet in red ($\Gamma_{in}$), retentate outlet in green ($\Gamma_{out,ret}$), and permeate outlet in yellow ($\Gamma_{out,perm}$).

In the present investigation, a membrane in a cross-flow operating condition is considered. The design is geared to a lab-scale membrane test cell setup. In such a setup, the height of the channel is very small in comparison to the other channel dimensions. It “*simulates the feed channel of spiral wound modules*.” [54]. To study the influence of the microcapsules on the flow, they need to be resolved in the numerical grid for the simulation. Thus, only a part of the membrane test cell can be considered in the simulation. Therefore, a representative elementary volume (REV) was selected. The schematic setup is shown in Figure 2. Its size was adjusted to include one hundred microcapsules ($N_{c}=100$) on the membrane surface in a regular arrangement as it is shown in Section 3.2. The diameter of one microcapsule is denoted by $d_{p}$. The inflow channel was chosen to be square. The dimensions of the reference area of the membrane surface were chosen as $A_{\mathrm{membrane}}=h_{c}\times l_{o}=10d_{p}\times 40d_{p}$ (to fit 5 microcapsules times 20 microcapsules with a regular spacing).

To achieve a fully-developed flow field above the membrane, a short channel part of length $10d_{p}$ was added directly after the inlet in front of the membrane area. The outlet length is $20d_{p}$. Thus, $l_{c}=70d_{p}$. Since a cross-flow configuration was considered, a typical REV with periodic boundary conditions could not be used (two outlets here). In the lateral direction, symmetric boundary conditions were prescribed. The height of the channel is given as $h_{c}$.

At the inlet $\Gamma_{in}$, the flow rate $Q_{in}=\left(u_{in}\;\cdot \;n_{in}\right)A_{in}$ was chosen such that an inlet velocity $u_{in}\;\cdot \;n_{in}=1m/s$ was achieved. At the permeate outlet $\Gamma_{out,perm}$, 10% of the fluid is drawn off with flow rate $Q_{out}=\left(u_{out}\cdot n_{out,perm}\right)A_{out}=-0.1\cdot Q_{in}$, i.e., 10% of the fluid is forced through the membrane. The remaining part of the fluid leaves the setup over the retentate outlet. Water was considered under standard conditions ($\rho_{f}=1000\;kg/m^{3}$ and μ=0.001Pas).

To specify if the flow is laminar or turbulent, the Reynolds number was computed. For the above setup, the Reynolds number in the inlet channel part was computed. Since symmetric boundary conditions were used, the setup was considered as an infinitely brought channel. Using the hydraulic diameter $D_{H}$, it is given in the above case as two times the height $D_{H}=2h_{c}$. Then, the Reynolds number is computed as:

(9) $$Re=\frac{\rho_{f}\left(u_{in}\times n_{in}\right)D_{H}}{\mu }=\frac{1000\;kgm^{3}\times 1\;ms\times 2h_{c}}{0.001\;\mathrm{Pa}s}=2\times 10^{6}h_{c}\;/m^{-1}.$$

For such a setup, the critical Reynolds number is stated as $Re_{crit}=2800$ [55]. In [56], it was stated that for a flow between parallel plates, the critical Reynolds number is 1500 if the distance between the plates is used as the characteristic length. To use the hydraulic diameter as above, this corresponds to a critical Reynolds $Re_{crit}=3000$.

Microcapsules with a diameter $d_{p}=130\;\mu m$ are intended to be placed on the membrane. Thus, the dimensions of the above setup can be deduced. As the height, $h_{c}=1.3\;mm$ was chosen; therefore, a rectangular channel was considered in the REV. The Reynolds number of the above setup was then $Re=2600<Re_{crit}$, and the flow could be considered to be laminar.

The membrane had a thickness L=130μm. Furthermore, the (effective) permeability of the membrane was chosen as $K=1\times 10^{-13}\;m^{2}$ and was, therefore, in a typical range for water filtration (see, e.g., [34]).

### 3.1. Microcapsule and Membrane Manufacturing

**Figure 3:**
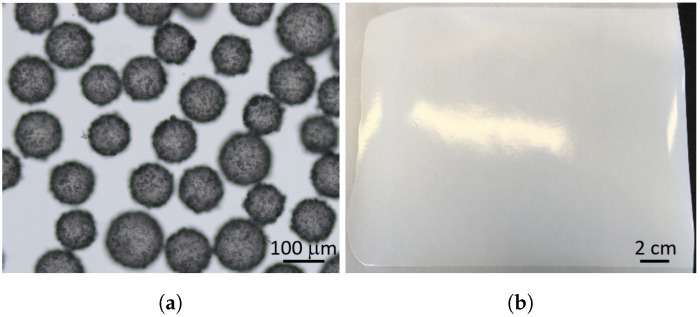
Components of the new membrane setup. (**a**) Spherical aminoplast microcapsules filled with eucalyptus oil. (**b**) Polymeric flat sheet ultrafiltration membrane made from polyacrylonitrile with nonwoven support.

Microcapsules composed of eucalyptus oil (content) and a highly cross-linked aminoplast polymer (shell material) were synthesized by an acid-catalyzed polycondensation reaction of a melamine-formaldehyde resin [57]. Eucalyptus oil is known for its natural antimicrobial properties and was used as a model biocide here [58]. The synthesized microcapsules had a spherical shape with a mean diameter ($D_{50}$) of 130μm, measured by laser diffraction. Several microcapsules are shown in Figure 3a.

Ultrafiltration flat sheet membranes were made of polyacrylonitrile (PAN) on a nonwoven support and formed by the nonsolvent-induced phase separation process (NIPS) [59]. The resulting membrane had an integral-asymmetric structure, i.e., anisotropic in the thickness direction but isotropic in the in-plane direction. As shown in Figure 3b, the pure PAN membrane for cross-flow filtration shows a glossy top layer with measured pore sizes of 20±2nm.

**Figure 4:**
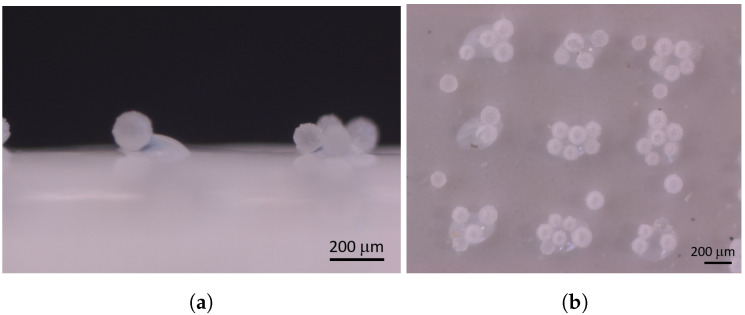
Optical microscopy images of the combined microcapsule membrane structure manufactured by the microdispensing approach (adhesive bonding). (**a**) Side view of microcapsules fixed on dispensed glue dots. (**b**) Top view on the membrane surface with the regular glue dot pattern and assemblies of microcapsules.

**Figure 5:**
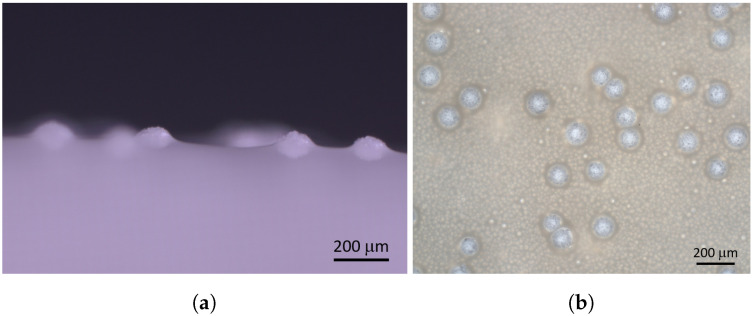
Optical microscopy images of the combined microcapsule-membrane structure manufactured by the in situ approach. (**a**) Side view of microcapsules embedded in the top layer of the membrane surface. (**b**) Top view of randomly distributed single microcapsules.

For the microcapsule fixation on the membrane surface, two strategies were developed. The testing procedure was assisted by simulation:

1. Fixation on glue dots: The ready membrane was modified with glue dots, onto which the microcapsules were trickled. With the help of a jet valve, adhesive dots were first applied to the membrane surface in a structured manner. The glue dots had a nearly hemispherical shape. The mean glue dot diameter and the glue dot height were measured using microscopy. The diameter was $d_{gd}=250\;\mu m$ and the height $h_{gd}=100\;\mu m$. The sometimes high impact energy of individual adhesive drops resulted in satellite drops, which were distributed and settled down between the regular printed structure. Their diameter was smaller than the diameter of the regular glue dots. In a second step, microcapsules were fixed by trickling onto the permanently adhesive dots. As shown in an optical microscopy image, this resulted in a cluster of several microcapsules on one glue dot (Figure 4). As visible, single microcapsules were located on the satellite drops.
  The interaction of the membrane surface and the glue can cause various phenomena that might lead to a change in the flow resistance. Gas flux experiments (dead end) on the real membrane with and without glue dots were performed. In comparison to the pure membrane without glue dots, the gas flux of membranes with glue dots was approximately proportional to the remaining free membrane surface left after the partial covering with glue dots. Therefore, phenomena such as damaged membrane structures or pore blocking due to dispensed glue outside of the glue dots could be excluded.
2. In situ microcapsule fixation: The microcapsules were fixed on the membrane surface by a new patent-registered in situ process [28], in which the microcapsules were trickled onto the top polymeric membrane layer before the precipitation process, leading to the polymer solidification, and pore formation was performed. The direct addition of microcapsules to the sticky polymer material resulted in a hemisphere geometry on the membrane surface, as is evident from the optical microscopy image (Figure 5).
  The advantage of this production process lies in the one-step synthesis procedure, which allows for the control of the number of microcapsules on the membrane surface. However, the production of structured microcapsule patterns could not be realized by this method so far (compare Figure 5b). The microcapsules were strongly fixed on the membrane surface since they were subsiding in the membrane layer and the nonwoven support. Furthermore, porometry measurements allowed studying the stability of the microcapsules. Experiments from 1 to 10 bar indicated a good microcapsule stability over the complete range.

The described and depicted microcapsule-membranes were the result of a continuing development process. When the study was started, it was assumed that microcapsules might be put in a perfect way on the membrane surface. In this idealization, single microcapsules are added to the surface of the membrane with a perfect point contact. In the classification of the two methods, this setup can be seen as an idealization. It reflects an infinitesimal glue dot, where only one microcapsule is fixed at (one glue dot at) the membrane surface.

### 3.2. Capsule Membrane Configurations

**Figure 6:**
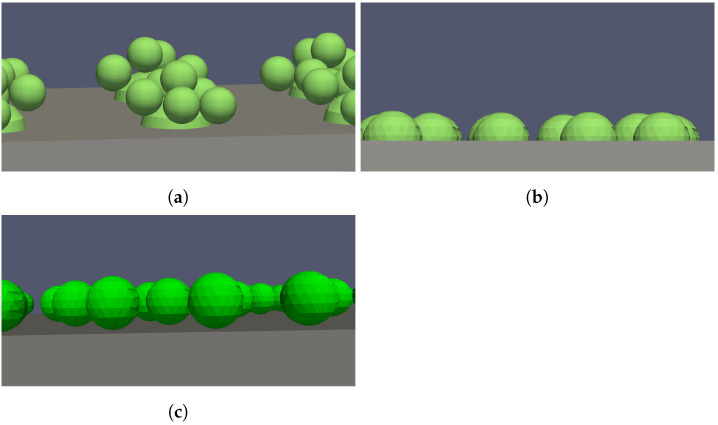
Overview of virtual geometries. (**a**) Side view of the virtual glue dots with microcapsules. (**b**) Side view of the virtual microcapsules partly subsiding in the membrane. (**c**) Side view of the virtual microcapsules with idealized point contact on the membrane surface.

The experimentally realized above-described microcapsule-membrane configurations were reproduced using virtual (or digital) geometries. Those geometries represent an idealized picture of the experimental results. Different variations in the arrangement of the capsules were tested using virtual geometries. Exemplary virtual geometries are shown in Figure 6. It was assumed that the glue dots always had an ellipsoidal shape and that the microcapsules were spherical with a constant diameter.

Comparing Figure 4a and Figure 6a shows that the virtual geometry mimics the real geometry well. In the virtual geometry, six microcapsules were added to one glue dot. As can be seen in Figure 4b, this was the maximum number of microcapsules that was achieved in the experiments. For the further simulations, the number of microcapsules was fixed to six for every glue dot, and a regular arrangement of glue dots was considered. Satellite drops were neglected. The distance between two dots (from midpoint to midpoint) was $\ell_{gd}=650\;\mu m$. The position of the microcapsules on the glue dots was varied. Using a random number generator, the positions of the microcapsules for each glue dot were determined. With the above-given distance between the glue dots, 16 glue dots could be positioned in the reference volume. Thus, in total, $N_{c}=96$ microcapsules were considered. Ten different random variants of the microcapsules on the glue dots were investigated.

**Figure 7:**
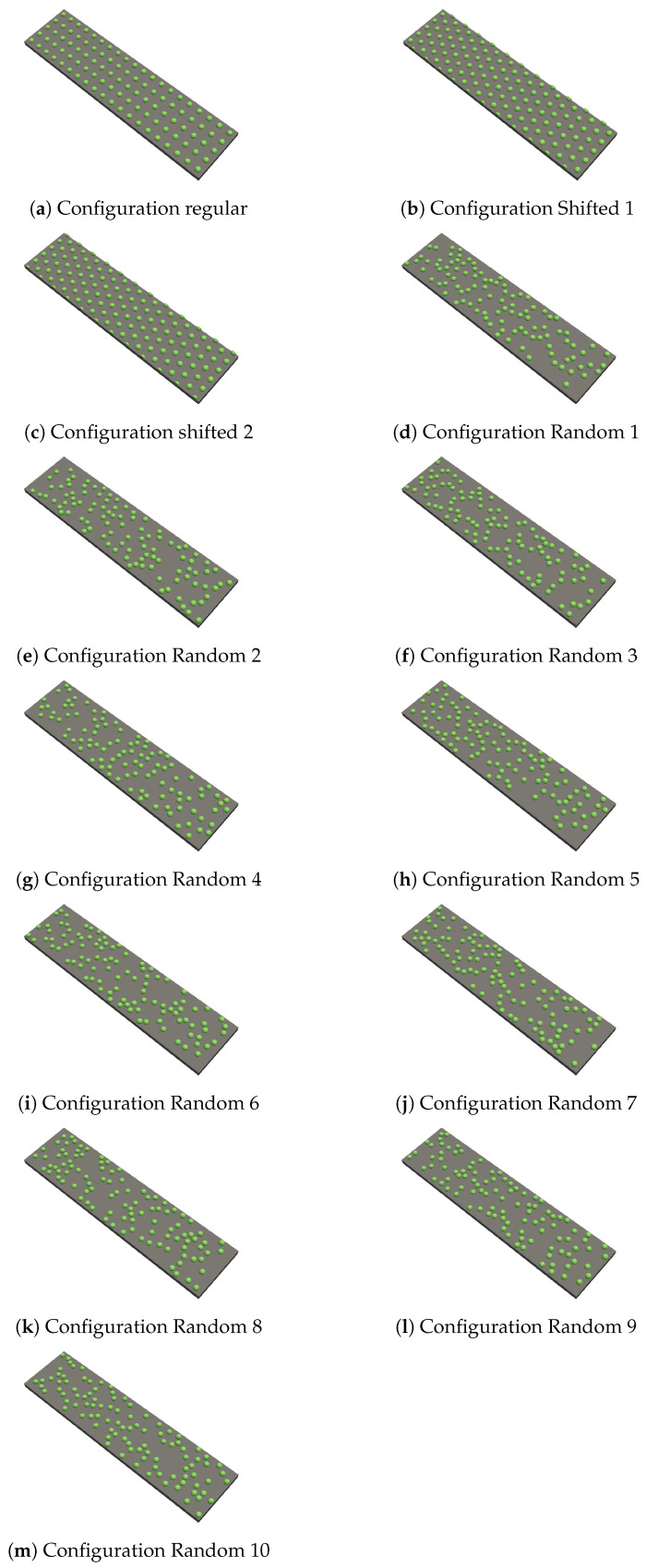
Overview of different microcapsule configurations.

For the method of adding the microcapsules during the manufacturing process of the membrane, the real and the virtual geometry are shown in Figure 5a and Figure 6b. In the real geometry, the penetration depth of the microcapsules was not completely uniform. However, for the virtual setup, it was considered uniform. Several placement patterns of the microcapsules were considered: three different configurations where the microcapsules were placed regularly and ten configurations where the microcapsules were placed randomly. The configurations are shown in Figure 7. In the first regular pattern, the microcapsules were placed on a square grid. In the other two regular configurations, the microcapsules were placed on a hexagonal grid. This means that in comparison to the regular placement, the microcapsules were shifted. The first one (Figure 7b) started with five microcapsules in the first row and was followed by four microcapsules and two half ones in the second row. This arrangement continued. In the second variant (Figure 7c), this arrangement was reversed. The first row started with four microcapsules and two half ones and continued with five microcapsules in the second row. The grid dimension was $2d_{p}$; thus, the distance between the single microcapsules was equal to $d_{p}$. In the hexagonal configurations (Figure 7b,c), the number of microcapsules was $N_{c}=115$. In all other configurations, $N_{c}=100$ microcapsules were used. The random placement of the microcapsules (Figure 7d–m) was comparable to the real geometry in Figure 5b.

In both methods, the membrane surface was reduced. Using the glue dots, the reduced membrane surface area $A_{gd}$ can be computed as:

$$A_{gd}=A_{\mathrm{membrane}}-16\;\cdot \;\pi \frac{d_{gd}^{2}}{4}=5.9746\;mm^{2},$$

where the surface might be additionally reduced by microcapsules on the glue dots, which are located at the edge of the glue dot. Thus, $A_{gd}/A_{\mathrm{membrane}}=0.88$. For the other method, the reduced membrane surface area $A_{m}$ is equal to:

$$A_{m}=A_{\mathrm{membrane}}-N_{c}\;\cdot \;\pi \frac{d_{p}^{2}}{4}=\left\{\begin{array}{cc} 5.4327\;mm^{2}, & N_{c}=100\\ 5.2336\;mm^{2}, & N_{c}=115 \end{array}\right..$$

In the first case, the membrane surface was reduced by 20% and in the second case by around 23%.

The virtual geometry of the idealized configuration is shown in Figure 6c. Here, the same configurations as for the previous one were considered. In reality, the membrane surface area would not be diminished, if a perfect point contact could be realized.

## 4. Numerical Results

For the simulation of the fluid flow, the software FiltEST was used [60]. The software is based on a finite volume discretization on a voxel grid [51,61]. The solver is based on a Chorin projection scheme, and the set of equations is solved until a steady-state solution is reached. To evaluate and compare the simulations, three different pressure criteria were investigated. For this purpose, the pressure values were averaged over a given plane, for example the inlet plane or the upstream side of the membrane:

1. Pressure difference between the inlet and retentate outlet $\Delta p_{c}$;
2. Pressure difference between the inlet and permeate outlet $\Delta p_{s}$;
3. Transmembrane pressure $\Delta p_{m}$.

Four different setups were investigated. Setup 0 considered a membrane without any modification by microcapsules, as shown in Figure 2. On the other hand, Setup 1 is the idealized one, where the microcapsules were positioned directly on the surface of the membrane. In Setup 2, the microcapsules were positioned on glue dots on the surface of the membrane. In setup 3, the microcapsules were partially subsided in the membrane surface (corresponding to adding microcapsules during the manufacturing process). When comparing the setups, also different arrangements of the microcapsules on the membrane surface were investigated. Using glue dots, the position of the microcapsules on the glue dots was varied. For Setups 1 and 3, the configurations are described in the previous section, and they are depicted in Figure 7.

Before presenting the comparison results, a grid study and a comparison of the numerical solution to approximate solutions from literature are shown.

### 4.1. Computational Grid Study and Approximate Solutions

To rate the quality of the numerical solution, the influence of the computational grid was investigated. As described above, a voxel grid was used, and therefore, the shape of the spheres and the glue dots could only be estimated. Therefore, the influence of the voxel size Δx on the solution was investigated. For the given values of Δx (see Table 1), the diameter of a microcapsule was resolved with either 4, 5, 8, 10, or 20 voxels, respectively. In Table 1, the three pressure criteria are shown for these discretizations. In Setup 2, the configuration Random 1, and in Setups 1 and 3, the configuration regular were used (for the details, see below).

One can see that the changes between the different voxel sizes (resolutions) were very small. The largest deviation can be observed in the pressure difference $\Delta p_{c}$ between the inlet and retentate outlet. This can be explained by the fact that the obstacles in the flow were differently resolved, and therefore, here, the influence of the voxel geometry was the largest. In comparison to the finest grid, the difference was the largest in Setup 2 (approximately 6%). The pressure difference $\Delta p_{s}$ was dominated by the transmembrane pressure. For Setup 0, the difference in $\Delta p_{m}$ was marginal, and for Setup 2 and Setup 3, it was approximately 2%. For Setup 1, it was around 3% Thus, also the difference in $\Delta p_{s}$ was in the same range. Therefore, a voxel size of Δx=13μm was chosen since, here, the computational efforts were accurate enough and the microcapsules were sufficiently resolved in the grid (approximately 10 voxels per microcapsule diameter). In this case, the number of voxels was equal to approximately 10,400,000. Therefore, the geometry was first discretized in a box with 700×100×185 = 12,950,000 voxels, and the solid voxels were removed since there was no flow.

Additionally, some parts of the pressure drop in Setup 0 can be estimated using approximate formulas for such a setup. The transmembrane pressure $\Delta p_{m}$ can be approximated using the integral form of Darcy’s law (Equation (4)). Using the above given values, this yields $\Delta p_{Darcy}=32.5\;k\mathrm{Pa}$. This is in good agreement with the above results.

The pressure drop in a rectangular channel of width *w* can be approximated using a formula stated in [62]. The formula reads:

(10) $$\Delta p_{\mathrm{channel}}=\frac{12u_{in}\mu l_{c}}{h_{c}^{2}}{\left[1-\frac{192h_{c}}{w\pi^{5}}\tanh\left(\frac{w\pi }{2h_{c}}\right)\right]}^{-1}.$$

As symmetry boundary conditions were used, the channel in the current setup can be considered as infinite wide. Taking the limit w→+∞, the formula simplifies similar to a 2D Poiseuille flow to:

(11) $$\Delta p_{\mathrm{channel}}=\frac{12u_{in}\mu l_{c}}{h_{c}^{2}}.$$

Comparing Darcy’s law (4) with Equation (11), one can define the dimensionless number $\chi =\frac{K^{-1}Lh_{c}^{2}}{l_{c}}$, which measures whether the channel or the transmembrane pressure drop is dominating. In our case, it was the transmembrane pressure drop ($\chi \approx 1.9\times 10^{9}$).

For $l_{c}=70d_{p}$ and $h_{c}=10d_{p}$, Equation (11) yields $\Delta p_{\mathrm{channel}}=64.62\;\mathrm{Pa}$. However, this is only an estimate, since this approximation does not consider the change in the flow conditions due to the flow through the porous membrane. In [63], an approximation of the pressure drop in a 2D channel with one porous wall was presented. It was based on a slip velocity derived in [64] and a similarity solution together with a perturbation analysis using the wall Reynolds number $Re_{w}$ defined as:

$$Re_{w}=\frac{\rho_{f}v_{w}h_{c}}{\mu },$$

where $v_{w}$ denotes the constant suction velocity along the porous wall. Using the methods developed in [65], the pressure drop can be computed as:

(12) $$\Delta p_{\mathrm{porous}\;\mathrm{channel}}=-\frac{C_{0}\mu l_{o}}{h_{c}^{2}}\left(u_{in}-\frac{v_{w}l_{o}}{2h_{c}}\right),$$

where $l_{o}$ is the length of the porous channel (see Figure 2) and $C_{0}$ an integration constant. In the case of a no-slip velocity condition on the porous wall (only normal velocity component), $C_{0}=-12$, in the case of a slip velocity $C_{0}=-12\frac{1+\phi }{1+4\phi }$ with $\phi =\frac{\sqrt{K}}{\alpha h_{c}}$. Here, α is a dimensionless constant depending on the pore size of the membrane [63]. In [63], α=0.1 was used. Updating the above approximation using no-slip conditions in the porous part and a reduced velocity in the outlet part of the channel yields $\Delta p_{\mathrm{channel}}=60.92\;\mathrm{Pa}$. If a slip velocity with α=0.1 is used, it reduces to $\Delta p_{\mathrm{channel}}=60.67\;\mathrm{Pa}$. However, this approximation is still below the value of the numerical simulation. Since for the derivation of the above formulas, a fully developed flow profile through the channel was assumed, this was not the case in the simulation. In the numerical case at hand, the flow started with a constant velocity and not with the developed velocity profile. Therefore, the additional pressure loss in the simulation was caused by the fact that the velocity had yet to develop. Due to this reason, the computed values for $\Delta p_{c}$ were higher. However, in cases in which the transmembrane pressure drop was dominating (as in the presented case), the solver gave reasonable results.

**Figure 8:**
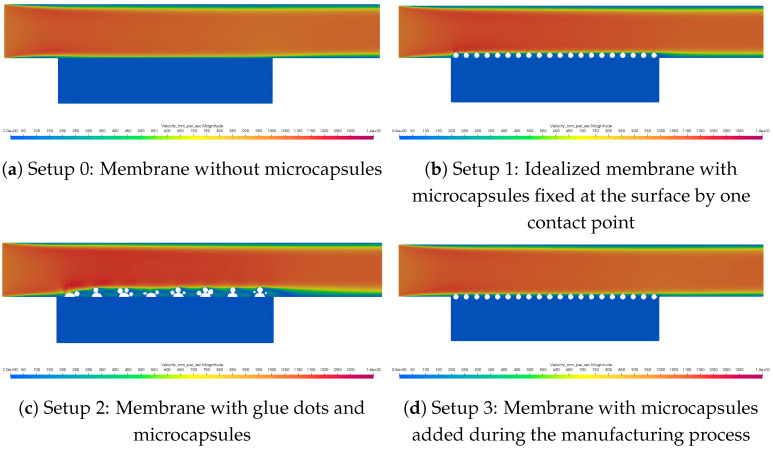
Overview of the velocity slices for the different setups.

In Figure 8, the comparison of the velocity fields of the four different setups is shown. Since the microcapsules were quite small in comparison to the channel height, the velocity field was only slightly affected. If the microcapsules were the only obstacles in the channel, the parabolic flow profile would be slightly shifted upwards. In the case of the glue dots, the influence was bigger since the dimensions of the obstacles were larger. Here, the shift in the parabolic flow profile was more obvious. Thus, in Setup 2, the channel pressure drop had its highest value, followed by Setup 1 and Setup 3, as is shown in Table 1.

### 4.2. Setup 1: Idealized Microcapsules on the Surface of the Membrane

For this setup, the configurations shown in Figure 7 were used. The microcapsules were shifted by their radius to touch the surface of the membrane (ideally at one point).

In Table 2, one can see that in comparison to Setup 0, the idealized configurations have only a small influence on the transmembrane pressure. The transmembrane pressure increases slightly since the surface of the membrane is reduced due to the voxelization of the microcapsules. Averaging the surface area of all considered cases, it is reduced by around 3.5%. A larger difference is visible in $\Delta p_{c}$ since the microcapsules are an additional obstacle in the top flow channel.

The variation in the pressure drop between the different configurations only varies slightly. This indicates that the positioning of the microcapsules on the membrane surface has no significant influence on the transmembrane and the channel pressure drop.

### 4.3. Setup 2: Microcapsules Positioned on Glue Dots on the Membrane Surface

First, it is investigated how the glue dots without microcapsules influence the membrane performance. Therefore, a simulation was performed and added as *without* in Table 3. Looking first at the transmembrane pressure, it was approximately 5% higher than in Setup 0 (see Table 1 for Δx=13μm). However, note that it was not increasing as might be expected by the reduction of the membrane surface. As shown before, the surface area in this configuration was reduced to approximately 88%. Thus, using Equation (4) with $A_{gd}$ and $Q_{out}$, the transmembrane pressure should increase by a factor of 1/0.88=1.14. This was not the case in the simulation since the membrane area below the glue dots was not blocked for fluid flow (compare also Figure 8c). Therefore, also the flow was entering this region. It has to be checked in the laboratory whether the region below the glue dots is completely or only partially blocked. This also depends on the membrane morphology. The channel pressure drop was smaller than in the previous setup since the glue dots alone were in comparison not as high as the microcapsules.

Adding the microcapsules to the glue dots and comparing the simulations to the result without microcapsules, one can see that the channel pressure $\Delta p_{c}$ was higher. This also transferred to $\Delta p_{s}$, whereas the transmembrane pressure $\Delta p_{m}$ was approximately the same. It was in some cases even slightly smaller.

The variation in the results for the different random configurations was below 1%. This indicates that the positions of the microcapsules on the glue dots only had a small influence on the different pressure drop criteria.

### 4.4. Setup 3: Microcapsules Added during the Manufacturing Process

For the simulation, the microcapsule placement patterns shown in Figure 7 were used. First, it was tested if it was enough to consider only the part of microcapsules that protruded from the surface of the membrane. Thus, a virtual geometry with hemispheres on the surface was designed. It was supposed that the reduction of the surface area was the main reason for the increase of the pressure drop. However, comparing the simulations with the complete microcapsules with the superficial ones, it was found that it is important to consider the complete geometry. As one can see in Table 4, the transmembrane pressure differs. For the hemisphere setup, it is in the same magnitude as for the setup with glue dots, but the transmembrane pressure drop of the setup considering the complete geometry is higher. Therefore, it was decided to consider the complete microcapsules.

In comparison to Setup 1, the increase in pressure drop was around 7% and in comparison to Setup 0 (see Table 1 for Δx=13μm), around 12%. One reason for the increase is the reduction of the membrane surface due to the subsidence of the microcapsules. In comparison to Setup 0, the surface area was diminished by around 20%. According to Equation (4) with $A_{m}$ and $Q_{out}$, this led to an increase of the transmembrane pressure by a factor of 1.24 (or 1.29 in the shifted arrangement). Similar to the explanation before, also in this setup, the region below the microcapsules was not completely blocked from the flow, and therefore, the increase in the transmembrane pressure was lower than expected.

The pressure drop within the top channel was lower in comparison to Setup 2. This was caused by the fact that only the upper part of the microcapsules was an obstacle in the channel and was, therefore, smaller than a glue dot. As in Setup 2, the variation of the positions of the microcapsules had only a small influence on the overall pressure drop.

As already mentioned in Section 3.1, the positions of the microcapsules can not be controlled in the manufacturing process. The simulations showed that the positions of the microcapsules had only a small influence on the overall pressure drop.

Comparing Setup 2 and Setup 3, the overall pressure drop $\Delta p_{s}$ was lower in Setup 2. Thus, in terms of pressure drop, gluing the microcapsules on the surface of the membrane is preferable to adding the microcapsules during the manufacturing process. As Setup 1 is an idealization of the gluing fixation, the overall pressure drop could be additionally reduced when the size of the glue dots can be reduced.

### 4.5. Velocity Gradients

In this subsection, the velocity changes close to the membrane surface are investigated. Therefore, the vorticity as defined in Equation (7) and the shear stress at the membrane surface as defined in Equation (8) were used. A similar study was performed in [41] for spacers. In Table 5, the mean and maximum vorticity magnitude over the complete domain are specified. For the shifted setup, the average of the two versions was taken. For the random configurations, only the mean values of the ten simulations are shown. As can be expected, the vorticity was lowest for the setup without any microcapsules. When adding microcapsules, the vorticity was increased since additional velocity gradients were introduced. The highest values can be observed for Setup 2 with microcapsules followed by Setup 1. This might be caused by the fact that putting multiple microcapsules on the gluing dots introduces a kind of *rough* surface that disturbs the flow.

**Figure 9:**
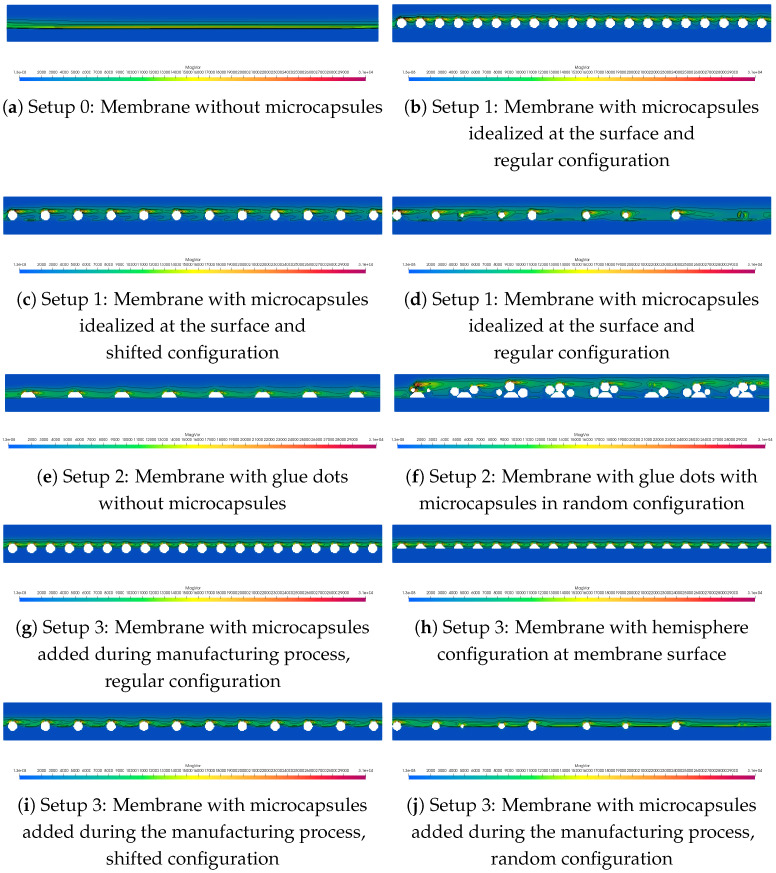
Overview of the vorticity contours for the different setups.

The most interesting region is the surface of the membrane where the microcapsules are located (since here, membrane biofouling will happen). Therefore, this region was extracted in the results, and slices through the extract were prepared. The contour lines of the vorticity magnitude in these slices are shown in Figure 9. The slice starts at the left with the beginning of the membrane and ranges to the end of the membrane surface. The vorticity was higher near the microcapsules due to the high-velocity variation (compare Figure 8). Whereas the contour lines of the vorticity are almost parallel to the membrane surface in the setup without microcapsules (Figure 9a), the microcapsules shift the contour lines up, and the highest values of the vorticity are obtained at the top side of the microcapsules. For regular configurations (Figure 9b,e,g), it can be observed that the patterns are repeated at each microcapsule, whereas for random configurations, the patterns differ. The reason for this might be the different spacing between the microcapsules. Comparing Setups 1 and 3 (e.g., Figure 9b,g), where the only difference is the height position of the microcapsules, the vorticity magnitude is higher in Setup 1.

**Figure 10:**
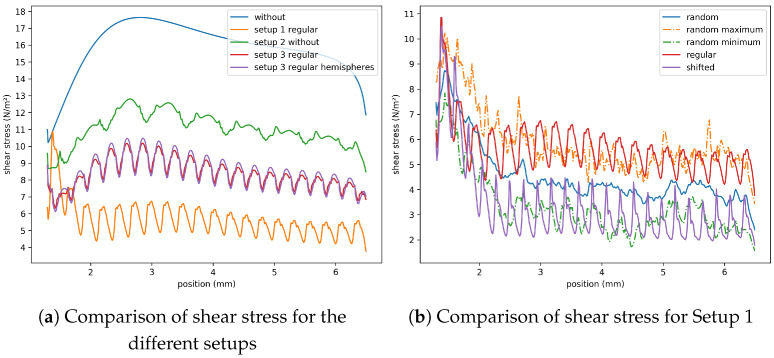

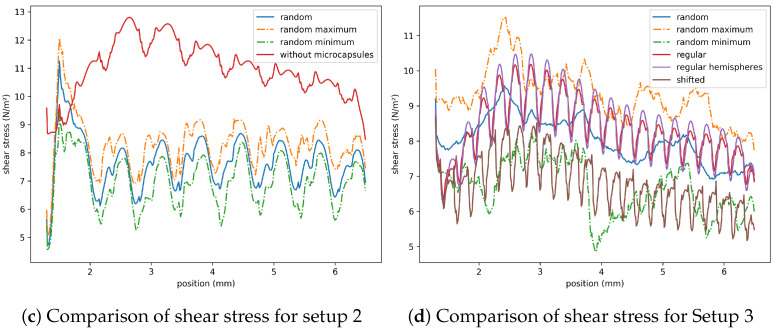
Distribution of the average shear stress at the top surface of the membrane.

The shear stress distribution at the top surface of the membrane is shown in Figure 10. To obtain these plots, a slice just above the membrane surface was extracted, and the shear stress was computed in every voxel. Then, the values were averaged along the $x_{2}$-direction. It can be seen as an area-weighted average of the shear stress at the membrane surface. Thus, the plots show the distribution of the average shear stress along the $x_{1}$-direction starting at the beginning of the membrane ($x_{1}=1.3\;mm$) to its end ($x_{1}=6.5\;mm$). For the random configurations, the results were averaged as before. As can be seen in Figure 10a, the shear stresses at the membrane surface reduce in all cases in comparison to the setup without microcapsules. The highest reduction is obtained for Setup 1. In the case of the regular placement of the microcapsules, the number of microcapsules can be clearly observed. The average shear stress increases at the microcapsules. Looking at the single setups separately, the following differences between the configurations can be observed. For Setup 1 (Figure 10b), the shifted setup has the lowest shear stress. This curve follows mainly the minimum of the random configurations, whereas the regular configuration follows the maximum of the random configurations. For Setup 2 (Figure 10c), the values for the glue dots with microcapsules are much smaller than for the glue dots without microcapsules. In Setup 3 (Figure 10d), the results are similar to Setup 1. However, contrary to the previous setups with microcapsules, the maximum of the shear stress is shifted to the right.

However, as can be seen in Figure 9, the zones of higher velocity gradients are shifted away from the membrane surface due to the obstacles. The velocity gradients (shear stresses) at the surface of the microcapsules are higher than those in the channel without microcapsules.

Summarizing the above results on the velocity gradients, the shear stress at the membrane surface is reduced in the setups with microcapsules. Looking at the vorticity, the mean and maximum vorticity are increased in these setups. However, the regions of higher vorticity are shifted away from the membrane surface. In comparison, Setup 2 leads to the highest velocity gradients and is therefore preferable to the other setups.

## 5. Conclusions and Outlook

In this article, two methods of membrane functionalization using the addition of microcapsules onto the membrane surface were discussed. Three main conclusions can be drawn from the simulations:

1. Gluing microcapsules yields a smaller pressure drop than the in situ fixation strategy;
2. The influence of the exact position of the microcapsules is smaller than the influence of the choice of the fixation strategy;
3. In comparison to the setup without microcapsules, the shear stress at the membrane surface is reduced in all cases, but the vorticity is increased and leads to a larger region of fluctuations of the velocity gradient. In comparison, also the gluing fixation is preferable.

In summary, the simulations showed that gluing microcapsules on the surface (Setup 2) should be preferred to adding the microcapsules during the manufacturing process (Setup 3). The dominating part of the pressure drop is in all cases the transmembrane pressure drop. For the gluing strategy, it is lower, and the reduction of the membrane surface is smaller in the considered setups. This also translates to the idealization in Setup 1, where single microcapsules are glued to the membrane surface.

Taking a look at the antibiofouling properties, a drawback of the gluing strategy is the larger distance between the single microcapsules. The distance is limited by the distance of the glue dots. At the moment, the size of the glue dots is limited to a minimal drop radius. This minimal radius is an interplay of the nozzle size and the glue used. Therefore, it has to be investigated if the idealized setup can be achieved. Additionally, other criteria such as stability against the flow have to be taken into account.

In general, the reduction of the membrane surface depends on the chosen method. Using the gluing fixation, the reduction of the membrane surface is mainly caused by the glue dots; however, the number of added microcapsules can be chosen independently and might only cause a small additional reduction of the membrane surface. On the other hand, for the in situ fixation, the reduction of the membrane surface is proportional to the number of microcapsules. Therefore, the decrease of the membrane surface might be better controllable using the in situ strategy. As shown above, the decrease of the membrane surface mostly influences the transmembrane pressure drop. Therefore, both methods allow selecting a suitable design when at least a minimal membrane surface area should be maintained.

In the next step, the release of biocide-loaded microcapsules and their impact on the biofouling behavior will be studied. Here, the influence of the permeate flux on the release process is important. Especially the distribution of the biocide over the membrane surface is an important indicator to find an appropriate configuration, and hence, the positioning of the microcapsules might be again important. Here, a balance between the reduction of the membrane surface by glue dots and microcapsules and the concentration of biocide has to be found.

Moreover, a possible combination of microcapsules and spacers might be performed. Spacers are widely used to promote turbulence [66]. This could help to distribute the biocide better over the surface of the membrane.

## Figures and Tables

**Table 1:** Comparison of pressure criteria for different grid resolutions.

	Δx (**μm**)	32.5	26	16.25	13	6.5
Setup 0	$\Delta p_{c}$ (kPa)	0.0842	0.0831	0.0818	0.0814	0.0803
	$\Delta p_{m}$ (kPa)	32.5005	32.5004	32.5002	32.5002	32.5002
	$\Delta p_{s}$ (kPa)	32.5540	32.5533	32.5532	32.5534	32.5541
Setup 1	$\Delta p_{c}$ (kPa)	0.1128	0.1136	0.1113	0.1101	0.1089
	$\Delta p_{m}$ (kPa)	33.1806	33.2857	32.8068	32.6741	32.5637
	$\Delta p_{s}$ (kPa)	33.3022	33.4118	33.9302	32.7950	32.6851
Setup 2	$\Delta p_{c}$ (kPa)	0.1431	0.1404	0.1373	0.1369	0.1350
	$\Delta p_{m}$ (kPa)	34.6393	34.4302	34.2542	34.2542	34.0858
	$\Delta p_{s}$ (kPa)	34.8117	34.5958	34.4183	34.3573	34.2517
Setup 3	$\Delta p_{c}$ (kPa)	0.0915	0.0949	0.0897	0.0888	0.0876
	$\Delta p_{m}$ (kPa)	37.2565	36.8097	36.9164	36.3879	36.0661
	$\Delta p_{s}$ (kPa)	37.3313	36.8944	36.9909	36.4606	36.1392

**Table 2:** Simulation results for idealized microcapsules on the surface of the membrane.

Configuration	$\Delta p_{c}$ (kPa)	$\Delta p_{m}$ (kPa)	$\Delta p_{s}$ (kPa)	Voxel Surface Area (mm^2^)
regular	0.1101	32.6741	32.7950	6.5403
Shifted 1	0.1144	32.7089	32.8477	6.4997
Shifted 2	0.1144	32.7989	32.8477	6.4997
Random 1	0.1142	32.6755	32.8037	6.5398
Random 2	0.1166	32.6754	32.8039	6.5400
Random 3	0.1138	32.6770	32.8065	6.5384
Random 4	0.1158	32.6776	32.8079	6.5378
Random 5	0.1139	32.6766	32.8068	6.5499
Random 6	0.1142	32.6737	32.8036	6.5418
Random 7	0.1145	32.6766	32.8068	6.5390
Random 8	0.1157	32.6745	32.8078	6.5410
Random 9	0.1154	32.6759	32.8084	6.5396
Random 10	0.1148	32.6776	32.8059	6.5378
mean Random	0.1149	32.6760	32.8061	6.5405

**Table 3:** Simulation results for positioning microcapsules on glue dots on the membrane surface.

Configuration	$\Delta p_{c}$ (kPa)	$\Delta p_{m}$ (kPa)	$\Delta p_{s}$ (kPa)	Voxel Surface Area (mm^2^)
without (only glue dots)	0.0920	34.2180	34.2893	5.9850
Random 1	0.1360	34.1929	34.3573	5.9868
Random 2	0.1365	34.1799	34.3443	5.9917
Random 3	0.1373	34.1845	34.3528	5.9870
Random 4	0.1363	34.1689	34.3317	5.9911
Random 5	0.1360	34.2210	34.3857	5.9740
Random 6	0.1376	34.1855	34.3493	5.9838
Random 7	0.1370	34.1831	34.3486	5.9911
Random 8	0.1369	34.1943	34.3611	5.9775
Random 9	0.1346	34.2080	34.3695	5.9785
Random 10	0.1359	34.2268	34.3919	5.9721
mean Random	0.1364	34.1945	34.3592	5.9834

**Table 4:** Simulation results for adding microcapsules during the manufacturing process.

Configuration	$\Delta p_{c}$ (kPa)	$\Delta p_{m}$ (kPa)	$\Delta p_{s}$ (kPa)	Voxel Surface Area (mm^2^)
regular hemispheres	0.0887	34.3220	34.3942	5.4756
regular	0.0888	36.3879	36.4606	5.4756
Shifted 1	0.0899	37.0562	37.1322	5.2850
Shifted 2	0.0899	37.0504	37.1264	5.2850
Random 1	0.0895	36.4520	36.5250	5.5028
Random 2	0.0904	36.4715	36.5441	5.4959
Random 3	0.0894	36.4930	36.5665	5.4918
Random 4	0.0901	36.4738	36.5275	5.4928
Random 5	0.0896	36.4593	36.5331	5.4957
Random 6	0.0896	36.5001	36.5733	5.5065
Random 7	0.0895	36.4886	36.5615	5.4918
Random 8	0.0899	36.4874	36.5614	5.4984
Random 9	0.0899	36.4757	36.5487	5.4912
Random 10	0.0897	36.4757	36.5481	5.4950
mean Random	0.0898	36.4777	36.5489	5.4962

**Table 5:** Vorticity results for all setups and configurations.

Setup	Configuration	Mean Vorticity Magnitude (/s)	Maximum Vorticity Magnitude (/s)
Setup 0	without	1053.2208	23,568.4141
Setup 1	regular	1152.4054	34,732.1717
	shifted	1133.4831	33,816.1440
	mean random	1133.7304	35,625.1141
Setup 2	without	1069.6653	31,373.9857
	mean random	1229.7577	44,146.4322
Setup 3	regular hemispheres	1060.9776	25,433.3640
	regular	1065.5370	25,451.6197
	shifted	1059.3224	25,834.9665
	mean random	1061.3415	30,031.0413

## Nomenclature

**Acronyms**
CFD	Computational fluid dynamics
REV	Representative elementary volume
PAN	Polyacrylonitrile
NIPS	Nonsolvent-induced phase separation
**Latin symbols**
*A*	Cross-sectional area
$D_{H}$	Hydraulic diameter
$d_{p}$	Diameter of the microcapsules
*K*	Permeability
*L*	Thickness of the membrane
$N_{c}$	Number of microcapsules
*n*	Outer unit normal vector
*p*	Pressure distribution
*Q*	Flow rate
Re	Reynolds number
*u*	Fluid velocity field
$x=(x_{1},x_{2},x_{3})$	Spatial coordinate
**Greek symbols**
Γ	Boundary of a domain
$\Delta p_{c}$	Pressure difference between the inlet and retentate outlet
$\Delta p_{s}$	Pressure difference between the inlet and permeate outlet
$\Delta p_{m}$	Transmembrane pressure
μ	Fluid viscosity
$\mu_{eff}$	Effective fluid viscosity
$\rho_{f}$	Fluid density
τ	Wall shear stress
Ω	Domain (subset of $R^{3}$)
ω	Vorticity
